# Air pollution and subclinical airway inflammation in the SALIA cohort study

**DOI:** 10.1186/1742-4933-11-5

**Published:** 2014-03-19

**Authors:** Mohammad Vossoughi, Tamara Schikowski, Andrea Vierkötter, Dorothea Sugiri, Barbara Hoffmann, Tom Teichert, Christian Herder, Thomas Schulte, Christian Luckhaus, Monika Raulf-Heimsoth, Swaantje Casjens, Thomas Brüning, Ursula Krämer

**Affiliations:** 1 IUF – Leibniz Research Institute for Environmental Medicine, Auf’m Hennekamp 50, Düsseldorf 40225, Germany; 2 Swiss Tropical and Public Health Institute, Socinstr. 57, Basel 4002, Switzerland; 3 University of Basel, Petersplatz 1, Basel 4003, Switzerland; 4 Institute for Clinical Diabetology, German Diabetes Center, Leibniz Center for Diabetes Research at Heinrich Heine University Düsseldorf, Auf’m Hennekamp 65, Düsseldorf 40225, Germany; 5 German Center for Diabetes Research (DZD), partner site Düsseldorf, Auf’m Hennekamp 65, Düsseldorf 40225, Germany; 6 Department of Psychiatry and Psychotherapy, Medical Faculty, Heinrich-Heine-University, Bergische Landstr. 2, Düsseldorf 40629, Germany; 7 Institute for Prevention and Occupational Medicine of the German Social Accident Insurance, Institute of the Ruhr-Universität Bochum (IPA), Bürkle-de-la-Camp-Platz 1, Bochum 44789, Germany; 8 Heinrich Heine University of Düsseldorf, Medical Faculty, Moorenstrasse 5, Düsseldorf 40225, Germany

**Keywords:** Particle exposure, Epidemiology, Inflammatory markers, Induced sputum, Exhaled breath condensate

## Abstract

**Background:**

The association between long-term exposure to air pollution and local inflammation in the lung has rarely been investigated in the general population of elderly subjects before. We investigated this association in a population-based cohort of elderly women from Germany.

**Methods:**

In a follow-up examination of the SALIA cohort study in 2008/2009, 402 women aged 68 to 79 years from the Ruhr Area and Borken (Germany) were clinically examined. Inflammatory markers were determined in exhaled breath condensate (EBC) and in induced sputum (IS). We used traffic indicators and measured air pollutants at single monitoring stations in the study area to assess individual traffic exposure and long-term air pollution background exposure. Additionally long-term residential exposure to air pollution was estimated using land-use regression (LUR) models. We applied multiple logistic and linear regression analyses adjusted for age, indoor mould, smoking, passive smoking and socio-economic status and additionally conducted sensitivity analyses.

**Results:**

Inflammatory markers showed a high variability between the individuals and were higher with higher exposure to air pollution. NO derivatives, leukotriene (LT) B_4_ and tumour necrosis factor-α (TNF-α) showed the strongest associations. An increase of 9.42 μg/m^3^ (interquartile range) in LUR modelled NO_2_ was associated with measureable LTB_4_ level (level with values above the detection limit) in EBC (odds ratio: 1.38, 95% CI: 1.02 -1.86) as well as with LTB_4_ in IS (%-change: 19%, 95% CI: 7% - 32%). The results remained consistent after exclusion of subpopulations with risk factors for inflammation (smoking, respiratory diseases, mould infestation) and after extension of models with additional adjustment for season of examination, mass of IS and urban/rural living as sensitivity analyses.

**Conclusions:**

In this analysis of the SALIA study we found that long-term exposure to air pollutants from traffic and industrial sources was associated with an increase of several inflammatory markers in EBC and in IS. We conclude that long-term exposure to air pollution might lead to changes in the inflammatory marker profile in the lower airways in an elderly female population.

## Background

It has been hypothesised that air pollution induced subclinical lung inflammation leads to a release of stress signals and of humoral mediators in the airways, which can spill over into the circulation and then contribute to functional impairment and ageing of other tissues and organs [1]. The first step of this cascade however, the association between long-term air pollution and subclinical inflammation of the airways, has rarely been investigated in the general elderly population so far. In a group of elderly subjects in Steubenville, Ohio, it could be shown that short-term exposure to PM_2.5_ may lead to airway inflammation as measured by the fraction of exhaled nitric oxide [2]. There are a few studies on susceptible or highly exposed cohorts. For example, in a group of waste handlers, daily exposure to bioaerosols over a short period of time increased the number of neutrophils and induced the secretion of interleukin (IL)-8 in the lower airways [3]. In a population of traffic policemen chronically exposed to traffic-related air pollution a statistically increased neutrophil cell count could be shown compared to a control group of healthy subjects without any exposure to traffic-related pollutants [4]. In a further investigation, living close to a major road was associated with neutrophilic bronchitis, asthma and decreased lung function in patients with airway diseases [5].

There is evidence that air pollution can induce a subsequent systemic inflammatory response. Alveolar macrophages play an important role in the association between the inflammatory process in the lung and the systemic inflammation because these cells are responsible for ingesting and clearing inhaled pollutants [1]. The interaction of macrophages with particulate matter leads to the increase of their phagocytic activity, oxidant production and release of other inflammatory markers such as tumour necrosis factor-α (TNF-α) [6,7]. However, other cells are also able to produce these mediators [1]. Additionally, it has been shown that a range of inhaled substances stimulate alveolar macrophages to produce proinflammatory cytokines such as IL-1, IL-6, and IL-8 and TNF-α [8-11]. Salvi et al. [12] showed a significant increase in neutrophils obtained from bronchial biopsies and also from peripheral blood 6 h after a short-term (1 h) exposure to diesel exhaust in a group of healthy volunteers. We are faced with a temporal association between air pollution, various markers of airway inflammation and subclinical inflammation. We aim to investigate the effect of both particulate matter (PM) and nitrogen dioxide (NO_2_) on a range of correlated inflammatory mediators of airway. Thus, it is of particular interest to assess not only the cell count, but also other inflammatory cytokines, which are induced by cell stimulation.

The above mentioned studies on the association of air pollution and subclinical airway inflammation focused on cohorts of either highly exposed or already diseased and presumably more susceptible subjects. However, the association of long-term exposure to air pollution has rarely been investigated in the general population of elderly women so far. Results from SALIA (Study on the influence of air pollution on lung function, inflammation and ageing),a cohort study of elderly German women, already demonstrated the link between long-term traffic-related air pollution and cardiovascular mortality [13], mild cognitive impairment [14], impaired glucose regulation [15], incidence of type 2 diabetes [16], accelerated skin ageing [17] and objectively measured chronic obstructive pulmonary disease (COPD) [18]. We hypothesise that in the SALIA study an air pollution-induced subclinical inflammation in the lung results in inflammatory mediators into the blood circulation, causing various downstream comorbidities. The SALIA study offers a good opportunity to investigate subclinical inflammation in the lung in a general population of elderly women. We therefore investigated the first step of this pathway namely the influence of long-term exposure to particulate matter and NO_2_ from traffic and industry on the level of inflammatory markers in exhaled breath condensate (EBC) and in induced sputum (IS) in a cross-sectional analysis including 402 elderly women of the population-based SALIA cohort in 2008/2009.

## Methods

### Study design and population

The SALIA study was initiated in the early 1980s by the State Government of North-Rhine Westphalia to investigate the health effects of air pollution exposure in women. The study population consists of women from the industrialized Ruhr Area in Germany (Dortmund, Duisburg, Herne, Gelsenkirchen, Essen) and two non-industrialized rural areas north of the Ruhr area (Borken, Dülmen). Men were not recruited because of the high occupational exposure of many men in this area, where coal mining and steel industry constituted the predominant sources of income in the time period before the baseline examination. Between 1985 and 1994 baseline examinations were conducted in 4874 women who were 55 years of age at time of recruitment. In 2006, a follow-up examination was conducted to assess the change in respiratory symptoms after a strong decline in concentrations of ambient air pollutants had taken place in the Ruhr Area. Women from Dülmen and Herne did not participate in the follow-up examination because of organisational restrictions in the local health departments. From 2116 (53% of surviving participants) women who responded to a self-administered postal questionnaire, 1639 women agreed to participate in further clinical examinations. 834 women from this group underwent a clinical examination at local study centres in 2008/2009. The present analysis is based on the first 402 women at the age of about 74 who were subjected to an extended collection of blood samples and analysis of inflammatory markers.

### Ethical approval

The ethical committee of the Ruhr University Bochum had favourably reviewed the SALIA Study in 2006 (Registration number: 2732). All women gave their written informed consent before the investigation.

### Air pollution assessment

We applied GIS (Geographic Information System) for the assessment of exposure. Using address coordinates of the participating women, exposure to fine particles, NO_2_ and traffic was estimated by three different methods:

First, data from monitoring stations maintained by the State Environment Agency covering the Ruhr area in an 8-km grid were used to reflect medium to large-scale spatial variation in air quality. Accordingly we defined long-term air pollution background exposure as five-year means of the years 2003–2007 of PM_10_ (PM with diameter ≤ 10 μm) and NO_2_. For this purpose we used the nearest measurement station to the women’s home address.

Second, we used land-use regression (LUR) models and data from a measurement campaign (2008/2009) gained in the framework of the EU-ESCAPE study (European Study of Cohorts for Air Pollution Effects) for the assessment of individual long-term exposure. Concentrations of pollutants were measured at 40 sites for NO_2_ and 20 sites for air-borne PM in the study area based on fourteen-day samples for each season and site. The validated land-use regression models were used to assign estimated NO_2_, PM_10_, PM_2.5_ and filter absorbance of PM_2.5_ (soot) concentrations to each individual’s residential address [19,20].

**Figure 1:**
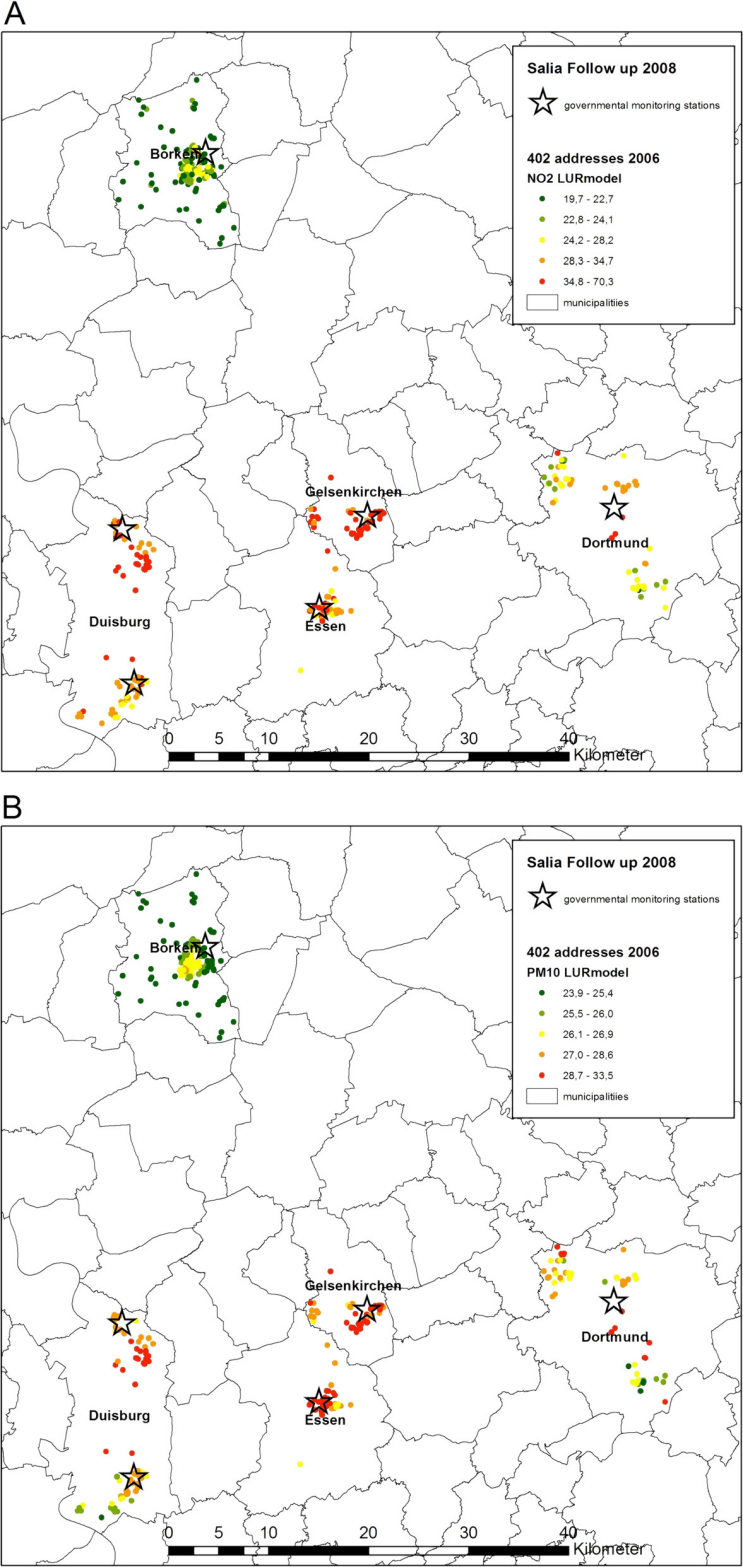
**Individual exposure to LUR-modelled NO**_**2 **_**(A) and PM**_**10 **_**(B).** Monitoring stations maintained by the State Environment Agency and the residential addresses of the participants with the corresponding exposure to the LUR-modelled NO_2_**(A)** and PM_10_**(B)** in the urban areas (Dortmund, Gelsenkirchen, Essen, Duisburg) and the rural area (Borken).

Third, traffic exposure was characterized by (1) the distance of the home address to the next major road, defined as ≥ 10,000 cars per day, and (2) the daily traffic volume within a 100 meters buffer around the home, calculated as the sum of the products of the number of vehicles from all roads with ≥ 5,000 cars per day multiplied with the street section length in the 100 m buffer. Figure 1 shows the monitoring stations maintained by the State Environment Agency and the residential addresses of the participants and the corresponding exposure to the LUR-modelled PM_10_ and NO_2_.

### Assessment of subclinical inflammation

All examinations were conducted according to standardized protocols. EBC was collected via “Eco-Screen” (VIASYS; Höchberg, Germany) from the participants. We analysed pH, the mediators leukotriene (LT) B_4_ and nitrate/nitrite, which reflect inflammation of the airways, and 8-isoprostane prostaglandin F_2α_ (8-iso PGF_2α_), which reflects oxidative stress. Women with acute infections of the respiratory tract were excluded from the examination. The measurement of pH was done via a pH-electrode. NO derivatives were measured by a colorimetric assay kit from Alexis (Cayman Chemicals; Grünberg, Germany) determining the total nitrate/nitrite concentration with a sensitivity of 5 μM. LTB_4_ and 8-iso PGF_2α_ were measured by a competitive enzyme immune assay (Assay Design, Ann Arbor, MI, USA).

After conducting the EBC procedure, participants inhaled vaporized isomolar saline solution for 10 minutes and were then asked to provoke coughing. IS was collected and processed according to Raulf-Heimsoth et al. [21] and then analysed for soluble inflammatory mediators and differential cell counts. After centrifugation, the cell-free supernatants were aliquoted, stored at −80°C until further analysis of soluble markers. The cell pellets were re-suspended and eosinophils, macrophages, neutrophils, epithelial cells and the total number of cells as the sum of these cells were determined. For differential cell counts cytospins were prepared, stained and counted by three independent observers. The same method as administered for the EBC examination was done to measure nitrate/nitrite and LTB_4_ in IS. IL-8, IL-1β and TNF-α were measured using ELISA technique described in detail in Raulf-Heimsoth et al. [21]. The Bradford protein assay was used to determine total protein content. In addition, matrix metalloproteinase-9 (MMP-9) was measured using a monoclonal “sandwich” enzyme immunoassay.

### Covariate assessment

We obtained information about a priori known potentially confounding factors from a standardized interview. We included smoking status (recorded as current, former and never smoking), current passive smoking at home, educational level defined as the maximum years of schooling of the woman or her husband as indicator of socio-economic status (low: less than 10 years, medium: 10 years, high: more than 10 years), age and indoor mould infestation which showed an association with outcomes or exposures. Heating with fossil fuels was selected as potential confounder but not included in the final model because it was neither associated with inflammatory outcomes nor with air pollution.

Additionally we used information about the mass of IS, participant’s moving history, urban/rural living, chronic airway diseases (COPD, asthma, and bronchitis) and season of the clinical examination in order to model the associations for subpopulations or with additional covariates as sensitivity analyses.

### Statistical methods

Continuous variables of inflammation showed a skewed distribution and a skewed residual distribution and therefore were log-transformed for analysis. If more than 50% of the values were below the detection limit or equal to zero, continuous variables of inflammation were transformed to binary variables using the detection limit or 0 as cut-off. These were LTB_4_ in EBC (83.3% below detection limit), and macrophages (56% = 0) and eosinophils (77% = 0) in IS. We hypothesise that exposure to air pollution leads to lower pH-values but higher values of other inflammatory markers. Since we expected pH to be inversely associated with air pollution, pH was transformed to 9-pH in order to obtain estimates in the same direction comparable with the estimates of other markers of inflammation. “9” was used for this transformation as the highest value for pH in our dataset. We applied multiple linear or logistic regression analysis to estimate the effect of air pollution and traffic exposure on inflammatory markers. Air pollution concentrations were entered as continuous terms except distance to a major road which was entered in categories (distance < 100 m, distance ≥ 100 m). After back-transformation of linear regression coefficients, percentage changes of inflammation variables and the corresponding 95% confidence intervals (95% CI) were presented for an interquartile range (IQR) increase in continuous air pollutants and for the binary variable “living close to major road”.

We estimated the associations in crude models (only adjusted for age) and in full models additionally adjusted for smoking status, passive smoking, educational level, exposure to indoor mould and age. The linearity of the exposure-response relationship was investigated via natural spline models in the statistical software R version 2.13.1 (package “splines”). All other statistical analyses were done with SAS version 9.2 (SAS Institute, Cary, NC).

### Sensitivity analyses

We investigated whether any effects were due to associations solely observed in specific subgroups of the women. Therefore we repeated the analysis in subgroups and present the effect estimates along with the original estimates in a series of figures. Additionally we included other covariates and tested different definitions of variables. These sensitivity analyses include:

(1) Exclusion of women with indoor mould infestation since mould infestation was already shown in SALIA to be a strong risk factor for the development of inflammatory reactions [22]. (2) Exclusion of currently and formerly smoking women. (3) Exclusion of women with diagnosed asthma or COPD or bronchitis at follow-up of study. (4) Exclusion of women with any change of their residential address since the baseline examination in order to test the stability of results. (5) TNF-α was additionally transformed into binary categories and logistic regression was conducted because just slightly more than 50% of the values (55.3%) were over the detection limit. (6) In an extended model we additionally adjusted for season of the clinical examination. (7) In a separate extended model we used an indicator for urban or rural residential area of women (Ruhr Area vs. Borken) and additionally adjusted the models for this binary variable in order to investigate, whether the estimates change when attaching more weight to the within-area air pollution contrast and whether unmeasured characteristics of rural and urban living confound our estimates. (8) We repeated the analyses with the continuous variable for proximity to the next major road instead of its binary variable in order to avoid information loss due to the dichotomisation. (9) Finally, we adjusted for mass of IS.

## Results

### Characteristics of participants

The characteristics of the 402 participants are shown in Table 1. The women had a mean age of about 74 (SD = 2.6) years and the majority of them were non-smokers. Women living in urban areas (N = 212) were more frequently smokers (4.7% vs. 1.1% for current smoking, 18.9% vs. 12.1% for former smoking) and more often exposed to indoor mould than women in the rural area (N = 190) (16% vs. 8.9%). Distributions of air pollution variables are presented in Table 2. Median levels of all air pollution variables were significantly higher for the urban areas compared to the rural area. The median of daily traffic load from major roads within 100 meters buffer is equal to zero because the majority of women were living further away than 100 meters from the next major road. Women lived on average 3.2 km (standard deviation = 2.3 km) away from the next monitoring station of State Environment Agency. The distributions of markers of inflammation in EBC and IS are presented in Table 3. The geometric means of the concentrations of most inflammatory markers in women from urban area were higher than those from women living in rural areas. These differences are most pronounced for NO derivatives, TNF-α, neutrophils and the total number of cells in sputum.

**Table 1:** Characteristics of the study population in rural and in urban areas in 2008/2009

	**Total area (N = 402)**	**Rural area (N = 190)**	**Urban area (N = 212)**	**p-Value**^**a**^
Age (means (sd))	74.1 (2.6)	74.0 (2.5)	74.2 (2.6)	0.3259
Years of schooling (%)				0.8655
<10	17.0	18.1	16.1	
=10	51.0	50.5	51.2	
>10	32.0	31.4	32.7	
Smoking (%)				0.0125
Current	3.0	1.1	4.7	
Former	15.7	12.1	18.9	
Never	81.3	86.8	76.4	
Current passive smoking (%)	38.6	36.8	40.3	0.4797
Exposure to indoor mould (%)	12.7	8.9	16.0	0.0330
Asthma^b^ (%)	9.7	8.5	10.9	0.4214
COPD^b^ (%)	3.2	3.1	3.3	0.9351
Chronic bronchitis^b^ (%)	11.0	11.6	10.5	0.7109
Change of home address (%)	14.2	11.1	17.0	0.0889

^a^p-Value of *t*-test or Wilcoxon test for the difference of means between rural and urban area.

^b^Reported as doctor-diagnosed diseases.

**Table 2:** **Distribution of PM, NO**_**2 **_**and traffic exposure in rural and in urban areas**

	**Total area (N = 402)**	**Rural area (N = 190)**	**Urban area (N = 212)**	**p-Value**^**e**^
**Variable**	**Median (IQR)**	**Median (IQR)**	**Median (IQR)**	
**Nearest monitoring stations (Five-year mean)**^**a**^				
NO_2_ [μg/m^3^]	30.8 (13.2)	20.2 (0)	33.4 (2.6)	< 0.0001
PM_10_ [μg/m^3^]	25.3 (3)	25.2 (0)	28.2 (3.4)	< 0.0001
**LUR-modelled exposure**^**b**^				
NO_2_ [μg/m^3^]	26.0 (9.42)	23.0 (2)	31.9 (10.3)	< 0.0001
PM_10_ [μg/m^3^]	26.4 (2.26)	25.6 (0.89)	27.7 (2.7)	< 0.0001
PM_2.5_ [μg/m^3^]	17.4 (2.06)	17.0 (0.42)	18.9 (1.7)	< 0.0001
PM_2.5_ absorbance [10^−5^ m^−1^]	1.38 (0.44)	1.20 (0.13)	1.62 (0.47)	< 0.0001
Traffic load^c^ [vehicle*km/day]	0 (990)	0 (0)	0 (1330)	0.0003
	%	%	%	
Distance < 100 m^d^	19.1	14.7	23.1	0.0331 ^f^

^a^Five-year mean of 2003 – 2007 from the nearest monitoring station of the State Environment Agency covering the area in an 8-km grid (1 station in Borken, 5 stations in Ruhr-Area).

^b^Land-use regression modelled exposure using data from a measurement campaign (2008/2009) gained in the framework of the EU-ESCAPE study for assessment of individual long-term exposure (Modelling based on the measurements from 40 stations for NO_2_ and 20 stations for PM).

^c^Traffic volume within a 100 m buffer around the home, calculated as the sum of the products of the number of vehicles from all roads with ≥ 5,000 vehicles per day multiplied with the street section length in the 100 m buffer.

^d^Distance of residential address < 100 m from major road with more than 10,000 vehicles per day.

^e^p-Value of Wilcoxon test for the difference of means between rural and urban area.

^f^p-Value of *χ*^2^-test for the difference between rural and urban area.

**Table 3:** Distribution of inflammatory markers of study population in rural and in urban areas in 2008/2009

	**Total area**		**Rural area**		**Urban area**		**p-Value**^**b**^
	**Geometric mean (gsd)**^**a**^	**N**	**Geometric mean (gsd)**^**a**^	**N**	**Geometric mean (gsd)**^**a**^	**N**	
**Markers in exhaled breath condensate**							
pH	7.1 (1.1)	381	7.1 (1.1)	185	7.1 (1.1)	196	0.854
NO derivatives [μM]	6.3 (1.8)	384	6.0 (1.8)	186	6.6 (1.9)	198	0.1379
LTB_4_ [pg/ml]^e^	8.9 (1.4)	372	8.7 (1.4)	181	9.0 (1.4)	191	0.2522
8-iso-PGF 2 α [pg/ml]	111 (1.9)	363	112 (1.9)	175	110 (2.0)	188	0.8583
**Soluble markers in induced sputum**							
Total protein content [μg/ml]	207 (2.1)	324	196 (2.1)	160	219 (2.0)	164	0.1687
IL-8 [pg/ml]^c^	974 (3.1)	318	991 (3.2)	158	959 (3.0)	160	0.7991
NO derivatives [μM]	14.0 (2.2)	324	12.5 (1.9)	160	15.8 (2.4)	164	0.0072
IL-1β [pg/ml]	15.1 (2.9)	320	15.8 (3.1)	159	14.5 (2.7)	161	0.5007
TNF-α [pg/ml]	1.8 (2.0)	320	1.6 (2.0)	158	2.0 (2.0)	162	0.0067
LTB_4_ [pg/ml]	642 (2.2)	324	608 (2.1)	160	679 (2.2)	164	0.1988
MMP-9 [ng/ml]^d^	94.0 (4.5)	279	84.0 (4.9)	135	104 (4.2)	144	0.2431
**Cells in induced sputum**							
Total number of cells (x10^5^)	14.5 (2.7)	324	13.0 (2.7)	160	16.2 (2.6)	164	0.0433
Macrophages^e^	0.1 (19.2)	321	0.1 (18.3)	158	0.1 (20.3)	163	0.4582
Neutrophils (x10^4^)^f^	15.6 (6.2)	321	12.2 (6.4)	158	19.8 (5.9)	163	0.0175
Eosinophils^e^	0.03 (9.1)	321	0.03 (8.0)	158	0.04 (10.1)	163	0.3601

^a^Geometric standard deviation, ^b^p-Value of *t*-test for the difference of geometric means between rural and urban area, ^c^Values > 20000 set to 20000, ^d^Values <2 set to 2, ^e^Transformed to binary variables because for LTB_4_ in EBC 83.3% of the values were below the detection limit and for macrophages 56% and for eosinophils 77% of the values were equal to zero, ^f^Values > 5x10^6^ set to 5x10^6^; Values < 1 set to 1 for the log-transformation.

### Results of main analyses

**Figure 2:**
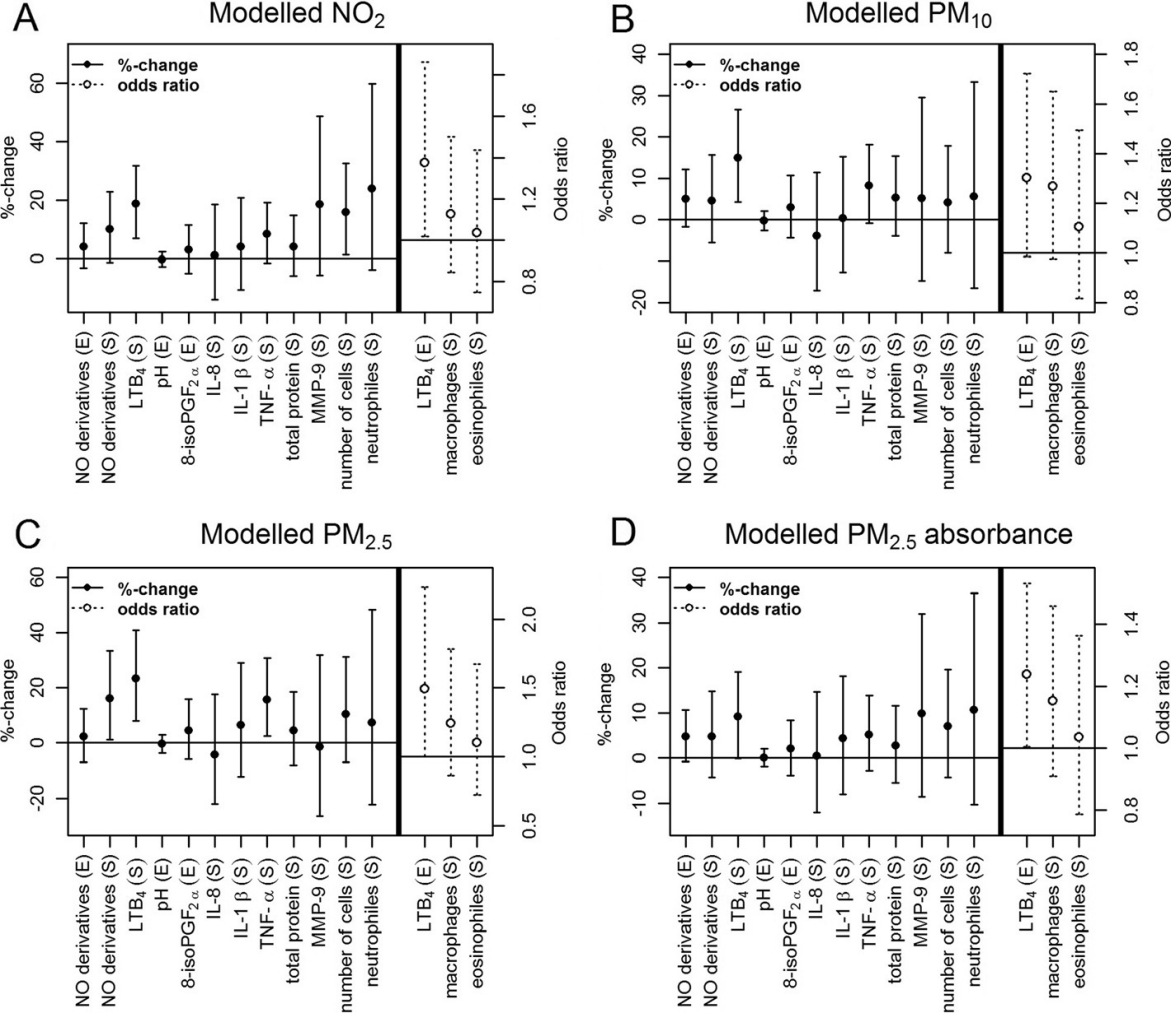
**Association of markers of inflammation with particles and NO**_**2**_**.** Percentage changes and odds ratios with the corresponding 95% confidence intervals for inflammatory markers in exhaled breath condensate (E)* and in induced sputum (S)* for an increase by one interquartile range (IQR) of land-use regression modelled NO_2_**(A)**, PM_10_ (B), PM_2.5_**(C)**, PM_2.5_ absorbance **(D)**, adjusted for age, smoking (current smoking, former smoking, never smoking), current passive smoking, indoor mould and socio-economic status by years of schooling. Number of women for each model: NO derivatives (E) =380, NO derivatives (S) =320, LTB_4_ (S) =320, pH (E) =377, 8-isoPGF_2α_ (E) =360, IL-8 (S) =314, IL-1β (S) =316, TNF-α (S) =316, Total protein (S) =320, MMP-9 (S) =275, number of cells (S) =321, neutrophils (S) =317, LTB_4_ (E) =369, macrophages (S) =317, eosinophils (S) =317. *Due to place restriction in the figures we did not use the same abbreviations for exhaled breath condensate (EBC) and induced sputum (IS) as stated in the text.

Figure 2 shows the association between inflammatory markers and individually estimated exposures for an increase by one IQR of air pollutant exposure in the adjusted model. Long-term exposure to modelled NO_2_ was significantly associated with LTB_4_ in EBC (odds ratio: 1.38, 95% CI: 1.02 - 1.86) as well as in IS (%-change: 19%, 95% CI: 7% - 32%). Additionally, an increase of 9.42 μg/m^3^ in modelled NO_2_ (IQR) was associated with a 15.9% (95% CI: 1.3% - 32.6%) increase in the total number of cells in IS. Modelled PM_10_, PM_2.5_ and PM_2.5_ absorbance showed the same pattern of effects. For example a 2 μg/m^3^ (IQR) increase in PM_2.5_ was associated with a 16.2% (95% CI: 1.1% - 33.5%) increase in NO derivatives in IS. Modelled PM_2.5_ was also significantly associated with TNF-α (%-change: 15.8%, 95% CI: 2.6% - 30.8%).

Individually estimated traffic volume showed positive associations with inflammatory markers. If traffic volume was increased by one IQR (990 vehicles*km/d), then the chance to have measurable LTB_4_ was 11% higher (95% CI: 1.1% - 21.5%). In contrast proximity to a major road did not show any consistent pattern of effects with markers of inflammation (Additional file 1: Figure S1).

Long-term exposure as measured at single monitoring stations was also positively associated with the inflammatory markers. Five-year mean of NO_2_ was significantly associated with LTB_4_ and NO derivatives in EBC and with NO derivatives and TNF-α in IS. Five-year mean of PM_10_ showed also significant association with LTB_4_ in EBC (Additional file 1: Figure S1). Air pollution variables showed mostly no associations with concentrations of 8-isoprostane and pH in EBC, and IL-8 and IL-1β in IS.

We conducted a stratified analysis for co-variables and for urban/rural living. We did not find any effect modification. Especially there was no indication that the effect was stronger in one area compared to the other one (data not shown). The crude associations differed only marginally from the fully adjusted associations (Additional file 1: Figure S2). We did not detect any significant deviations from linearity for the exposure-response associations (data not shown).

### Results of sensitivity analyses

Exclusion of subpopulations or extension of models with additional adjustment for covariables as sensitivity analyses did not change the results relevantly (Additional file 1: Figure S3-S8). Adjustment for urban/rural living attenuated the associations for NO derivatives and TNF-α in sputum and LTB_4_ in EBC. Estimates were attenuated for modelled NO_2_ and modelled PM_2.5_ after adjustment for urban/rural living. (Additional file 1: Figure S9). Effects for TNF-α were in the same direction after transforming the continuous variable into a binary one (Additional file 1: Figure S10). Finally, the associations with distance to major road - modelled as a continuous variable - point into the same direction as the results for the binary variable (Additional file 1: Figure S11).

## Discussion

In this analysis within the SALIA study we found that long-term exposure to air pollutants from traffic and industrial sources was associated with an increase of several inflammatory markers in EBC and in IS. Inflammatory markers showed a high interindividual variability. Amongst others the already known associations between these markers and smoking and mould infestation contributed to this variability [22]. These markers were increased when air pollution was higher. Significant associations of long-term air pollution were shown for LTB_4_ and NO derivatives in EBC and for total number of cells, NO derivatives, TNF-α and LTB_4_ in IS. The most consistent associations were found for LTB_4_ in EBC and changed between 11% (95% CI: 1.1% - 21.5%) for traffic volume up to 104% (95% CI: 27.7% - 226%) for five-year mean of NO_2_, respectively. LTB4 is a potent chemoattractant of neutrophils and was shown to contribute significantly to neutrophil influx into the airway in COPD patients [23]. IL-8, IL-1β, pH and 8-isoprostane were not associated with air pollution.

Few studies before have investigated long-term exposure to air pollution and markers of local pulmonary inflammation. In patients with COPD, neutrophils, LTB_4_, IL-8, macrophages, MMP-9 and TNF-α in airways were shown to be increased in several studies [24-28], however little is known about their associations with long-term air pollution in elderly women from the general population. We found a statistically significant association of PM_2.5_ with NO derivatives in IS and of the five-year mean NO_2_ exposure with NO derivatives in EBC. This observation of an up-regulation of inflammatory activity by long-term exposure to air pollution is supported by a study on elderly subjects in Steubenville, Ohio, which demonstrated an association between long-term exposure to PM_2.5_ and the fraction of exhaled nitric oxide (NO) [2].

Modelled long-term NO_2_ exposure was associated with total cell count in IS, however none of the exposures were clearly associated with neutrophil counts in sputum. This is in contrast to a case–control study on policemen [4], which found a statistically significant increase in the percentage of neutrophils in traffic policemen (median = 65, IQR = 13.5) compared to healthy subjects (median = 40.5, IQR = 9.5; p < 0.01) after a long-term exposure to traffic pollutants. Similarly to our study, they did not find any association of air pollutants with eosinophils, although our analysis is based on a binary variable for eosinophils indicating values below or above the detection limit.

Adjustment for urban/rural living attenuated the associations particularly for NO derivatives and TNF-α in sputum and LTB_4_ in EBC. This might be due to the lack of exposure contrasts within the respective areas of residence. We did not include urban/rural living into the main model in order to attach more weight to the between-area air pollution contrast. It is therefore possible, that other, unmeasured characteristics of rural and urban living could have confounded our estimates. Extensive sensitivity analyses with additional covariates however did not yield qualitatively different results.

Schikowski et al. [18] showed that long-term exposure to high concentrations of air pollutants at baseline of the SALIA study were associated with reduced lung function and prevalence of COPD. Therefore it was not clear whether an association of air pollution with local pulmonary inflammatory markers demonstrated a direct effect of current air pollution at follow-up of the SALIA study (2008) or an indirect effect of air pollution at baseline (1985–1994) mediated by a long-lasting effect through established lung diseases with probable inflammation. Therefore we excluded women with diagnosed bronchitis or asthma or COPD. This exclusion did not change our results. Therefore we consider our results as indication of an effect of current air pollution on subclinical pulmonary inflammation.

The strength of the study is that SALIA is a population-based cohort of elderly women (not a cohort of either highly exposed or diseased and more susceptible subjects) with a detailed exposure assessment and an in-depth characterization of subclinical pulmonary inflammation. We assessed not only the LUR-modelled air pollution but also air pollution at single monitoring stations in order to consider the long-term spatial background air pollution in addition to the individually modelled exposure. Different findings for LUR-modelled and corresponding five-year means of pollutants, which are most pronounced for LTB_4_ in IS, might be due to the better spatial resolution of the LUR-modelled exposures. Traffic related air pollution is better described with the LUR-models. However, the effects for five-year means of exposures are in the same direction as the effects for LUR-modelled exposure. One limitation of the current study is that our study was based on a subset of the cohort population which attended the follow-up examination in 2008/2009 at a mean age of 74 years. It is therefore possible that the results are attenuated by a healthy survivor effect.

## Conclusions

In this population-based study we could show a cross-sectional association between long-term exposure to air pollutants and concentration of several inflammatory markers in fluids collected from the lower respiratory tract in an elderly female population. We conclude that long-term exposure to air pollution might lead to changes in the inflammatory marker profile in the lower airways.

## Abbreviations

COPD: Chronic obstructive pulmonary disease; EBC: Exhaled breath condensate (“E” is used in the figures due to place restriction); ESCAPE: European study of cohorts for air pollution effects; GIS: Geographic information system; IL-1β: Interleukin-1β; IL-8: Interleukin-8; IQR: Interquartile range; IS: Induced sputum (“S” is used in the figures due to place restriction); LTB4: leukotriene (LT) B_4_; LUR: Land-use regression; MMP-9: Matrix metalloproteinase-9; NO2: nitrogen dioxide; OR: Odds ratio; PM: Particulate matter; PM10: Particulate matter with diameter ≤ 10 μm; PM2.5: Particulate matter with diameter ≤ 2.5 μm; SALIA: Study on the influence of air pollution on lung function inflammation and ageing; TNF-α: Tumour necrosis factor-α; 8-iso PGF2α: 8-isoprostane prostaglandin F_2α_; 95% CI: 95% confidence interval

## Competing interests

The authors report no competing interest.

## Authors’ contributions

Study idea and design: UK, TS, AV, MRH. Statistical analysis: MV. Interpretation of results: MV, TS, AV, BH, TT, CH, ThS, CL, MRH, SC, TB, UK. Exposure modelling: DS. Assessment of subclinical inflammation: MRH, SC. All authors participated in manuscript preparation. All authors read and approved the final manuscript.

## Supplementary Material

Additional file 1: Figure S1 Association of markers of inflammation with particles, NO_2_ and traffic exposure. **Figure S2.** Association of markers of inflammation with particles and NO_2_ (crude model vs. adjusted model). **Figure S3.** Association of markers of inflammation with particles and NO_2_ (subpopulation without indoor mould vs. total population). **Figure S4.** Association of markers of inflammation with particles and NO_2_ (subpopulation without current and former smoking vs. total population). **Figure S5.** Association of markers of inflammation with particles and NO_2_ (subpopulation without COPD, asthma and bronchitis vs. total population). **Figure S6.** Association of markers of inflammation with particles and NO_2_ (subpopulation without change of residential addresses vs. total population). **Figure S7.** Association of markers of inflammation with particles and NO_2_ (model additionally adjusted for season of examinations vs. model not adjusted for season). **Figure S8.** Association of markers of inflammation with particles and NO_2_ (model additionally adjusted for mass of induced sputum vs. model not adjusted for mass of induced sputum). **Figure S9.** Association of markers of inflammation with particles and NO_2_ (model additionally adjusted for urban/rural living vs. model not adjusted for urban/rural living). **Figure S10.** Association of a) continuous variable for TNF-α and b) binary variable for TNF-α with particles, NO_2_ and traffic exposure. **Figure S11**. Association of markers of inflammation with distance to major road (continuous variable for distance vs. binary variable for distance). Click here for file
