# Supplementary material for: Air pollution and subclinical airway inflammation in the SALIA cohort study
Source: Immun Ageing. 2014 Mar 19;11:5. doi: 10.1186/1742-4933-11-5 (PMC4000047; doi:10.1186/1742-4933-11-5)
Supplement: Additional file 1: Figure S1 — Association of markers of inflammation with particles, NO2 and traffic exposure. Figure S2. Association of markers of inflammation with particles and NO2 (crude model vs. adjusted model). Figure S3. Association of markers of inflammation with particles and NO2 (subpopulation without indoor mould vs. total population). Figure S4. Association of markers of inflammation with particles and NO2 (subpopulation without current and former smoking vs. total population). Figure S5. Association of markers of inflammation with particles and NO2 (subpopulation without COPD, asthma and bronchitis vs. total population). Figure S6. Association of markers of inflammation with particles and NO2 (subpopulation without change of residential addresses vs. total population). Figure S7. Association of markers of inflammation with particles and NO2 (model additionally adjusted for season of examinations vs. model not adjusted for season). Figure S8. Association of markers of inflammation with particles and NO2 (model additionally adjusted for mass of induced sputum vs. model not adjusted for mass of induced sputum). Figure S9. Association of markers of inflammation with particles and NO2 (model additionally adjusted for urban/rural living vs. model not adjusted for urban/rural living). Figure S10. Association of a) continuous variable for TNF-α and b) binary variable for TNF-α with particles, NO2 and traffic exposure. Figure S11. Association of markers of inflammation with distance to major road (continuous variable for distance vs. binary variable for distance). [file 1742-4933-11-5-S1.doc]

**Additional file 1:**


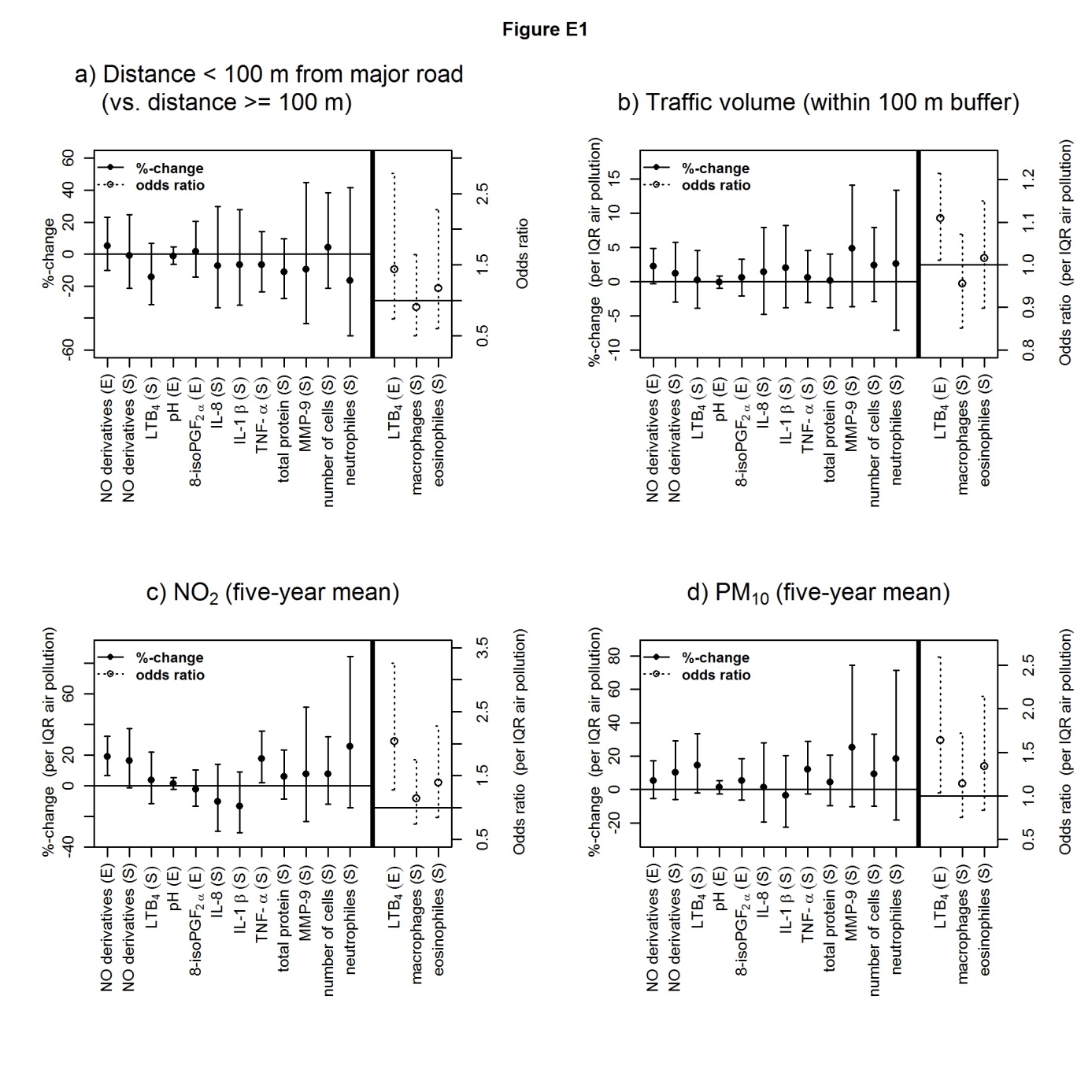


**Figure S1: Association of markers of inflammation with particles, NO_2_ and traffic exposure.**

Percentage changes and odds ratios with the corresponding 95% confidence intervals for inflammatory markers in exhaled breath condensate (E)* and in induced sputum (S)* for living close to major road (a) and for an increase by one interquartile range (IQR) of traffic volume (b) and five-year mean of NO_2_ (c) and PM_10_ (d) adjusted for age, smoking (current smoking, former smoking, never smoking), current passive smoking, indoor mould and social status by years of schooling.

Number of women for each model: NO derivatives (E) =380, NO derivatives (S) =320, LTB_4_ (S) =320, pH (E) =377, 8-isoPGF_2α_ (E) =360, IL-8 (S) =314, IL-1β (S) =316, TNF-α (S) =316, Total protein (S) =320, MMP-9 (S) =275, number of cells (S) =321, neutrophils (S) =317, LTB_4_ (E) =369, macrophages (S) =317, eosinophils (S) =317.

* Due to place restriction in the figures we did not use the same abbreviations for exhaled breath condensate (EBC) and induced sputum (IS) as stated in the text.


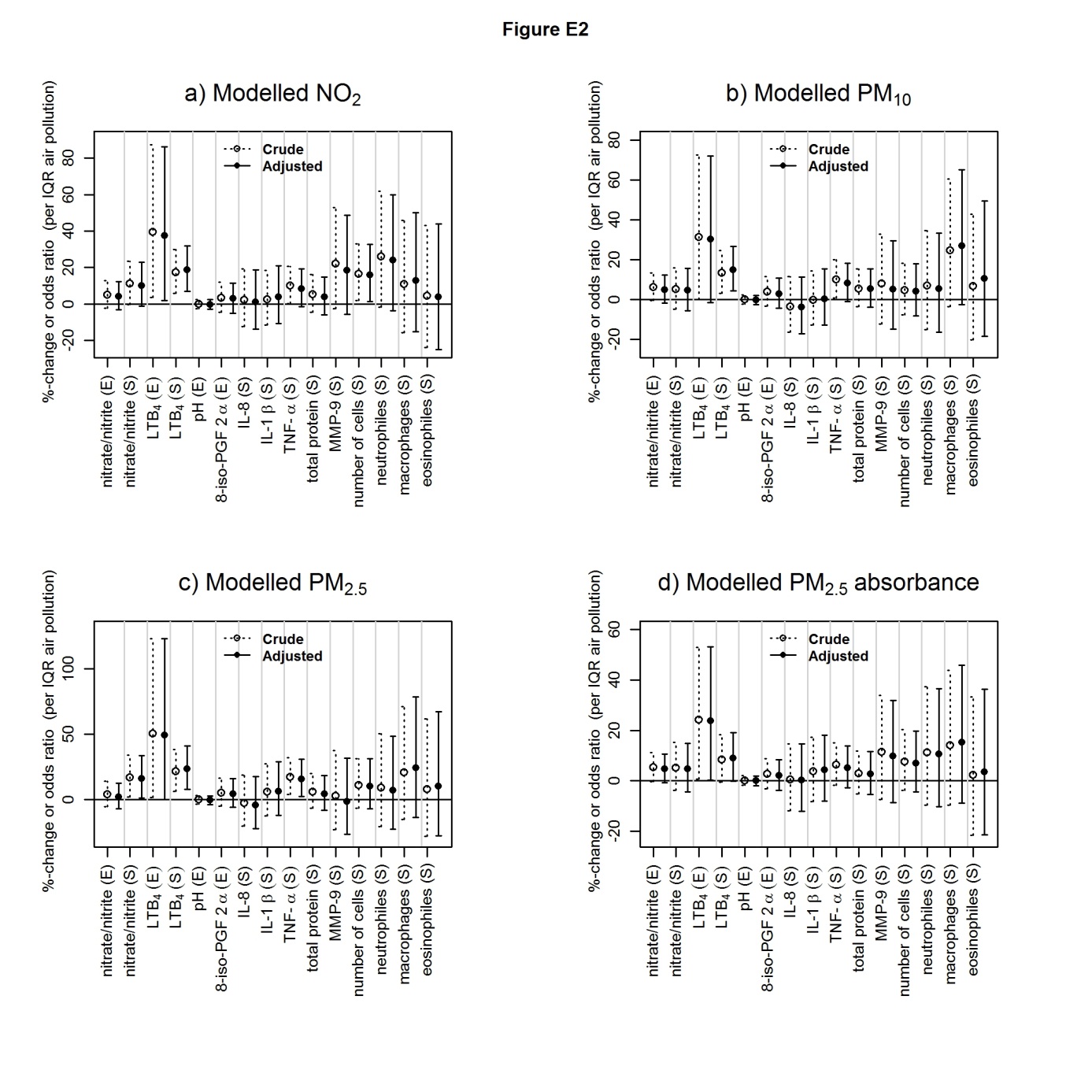


**Figure S2: Association of markers of inflammation with particles and NO_2_ (crude model vs. adjusted model).**

Percentage changes and odds ratios with the corresponding 95% confidence intervals for inflammatory markers in exhaled breath condensate (E)* and in induced sputum (S)* for an increase by one interquartile range (IQR) of land-use regression modelled NO_2_ (a), PM_10_ (b), PM_2.5_ (c) and PM_2.5_ absorbance (d).

Crude model included only age as co-variable. Adjusted model included age, smoking (current smoking, former smoking, never smoking), current passive smoking, indoor mould and social status by years of schooling as co-variables.

Number of women for each model: NO derivatives (E) =380, NO derivatives (S) =320, LTB_4_ (S) =320, pH (E) =377, 8-isoPGF_2α_ (E) =360, IL-8 (S) =314, IL-1β (S) =316, TNF-α (S) =316, Total protein (S) =320, MMP-9 (S) =275, number of cells (S) =321, neutrophils (S) =317, LTB_4_ (E) =369, macrophages (S) =317, eosinophils (S) =317.

* Due to place restriction in the figures we did not use the same abbreviations for exhaled breath condensate (EBC) and induced sputum (IS) as stated in the text.


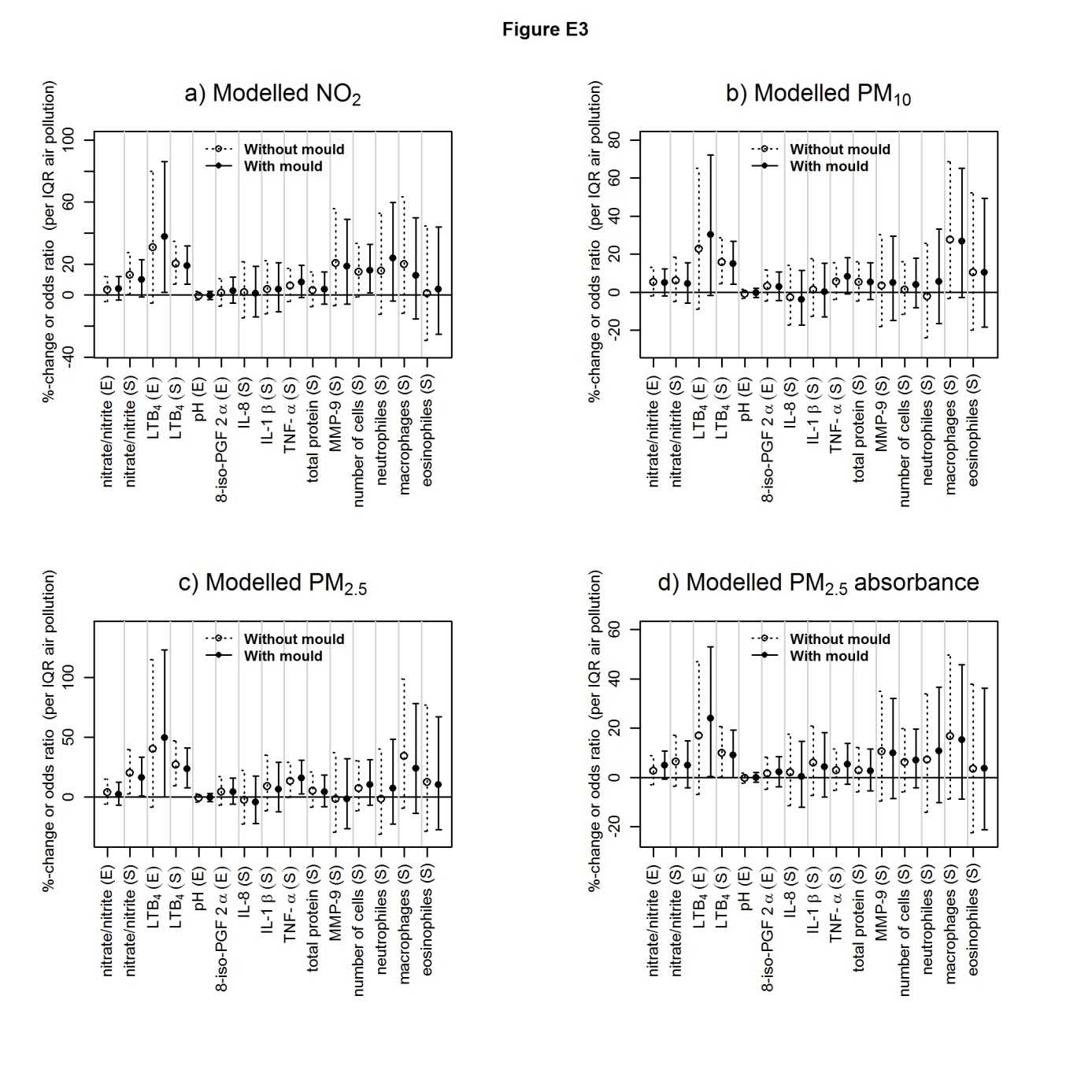
**Figure S3: Association of markers of inflammation with particles and NO_2_ (subpopulation without indoor mould vs. total population).**

Percentage changes and odds ratios with the corresponding 95% confidence intervals for inflammatory markers in exhaled breath condensate (E)* and in induced sputum (S)* for an increase by one interquartile range (IQR) of land-use regression modelled NO_2_ (a), PM_10_ (b), PM_2.5_ (c) and PM_2.5_ absorbance (d) adjusted for age, smoking (current smoking, former smoking, never smoking), current passive smoking, indoor mould and social status by years of schooling.

Number of women without indoor mould vs. total population for each model: NO derivatives (E) =330 vs. 380, NO derivatives (S) =274 vs. 320 , LTB_4_ (S) =274 vs. 320, pH (E) =327 vs. 377, 8-isoPGF_2α_ (E) =310 vs. 360, IL-8 (S) =268 vs. 314, IL-1β (S) =270 vs. 316, TNF-α (S) =271 vs. 316, Total protein (S) =274 vs. 320, MMP-9 (S) =230 vs. 275, number of cells (S) =274 vs. 321, neutrophils (S) =271 vs. 317, LTB_4_ (E) =319 vs. 369, macrophages (S) =271 vs. 317, eosinophils (S) =271 vs. 317.

* Due to place restriction in the figures we did not use the same abbreviations for exhaled breath condensate (EBC) and induced sputum (IS) as stated in the text.


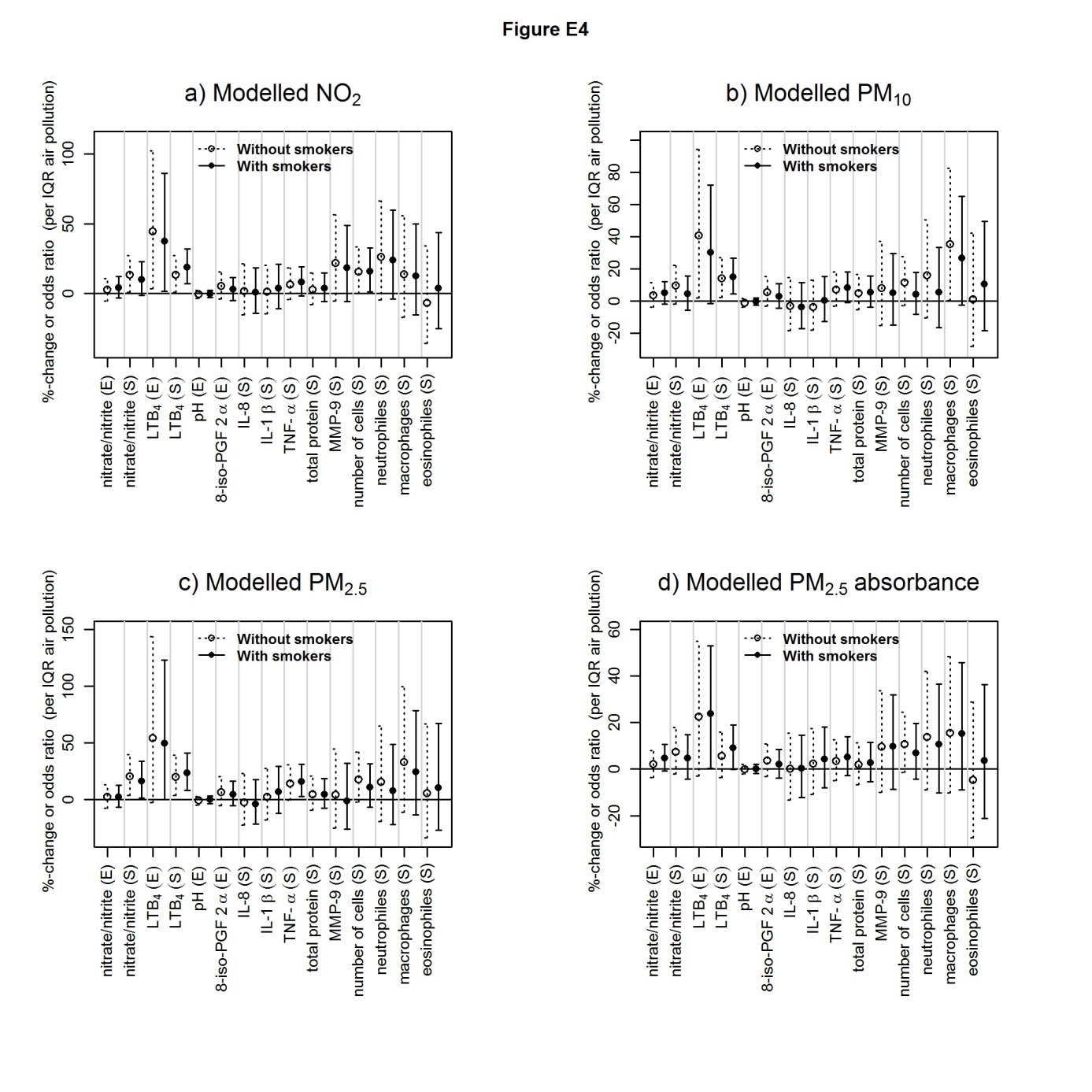
**Figure S4: Association of markers of inflammation with particles and NO_2_ (subpopulation without current and former smoking vs. total population).**

Percentage changes and odds ratios with the corresponding 95% confidence intervals for inflammatory markers in exhaled breath condensate (E)* and in induced sputum (S)* for an increase by one interquartile range (IQR) of land-use regression modelled NO_2_ (a), PM_10_ (b), PM_2.5_ (c) and PM_2.5_ absorbance (d) adjusted for age, smoking (current smoking, former smoking, never smoking), current passive smoking, indoor mould and social status by years of schooling.

Number of women without current and former smoking vs. total population for each model: NO derivatives (E) =308 vs. 380, NO derivatives (S) =268 vs. 320 , LTB_4_ (S) =268 vs. 320, pH (E) =305 vs. 377, 8-isoPGF_2α_ (E) =292 vs. 360, IL-8 (S) =263 vs. 314, IL-1β (S) =266 vs. 316, TNF-α (S) =264 vs. 316, Total protein (S) =268 vs. 320, MMP-9 (S) =228 vs. 275, number of cells (S) =268 vs. 321, neutrophils (S) =265 vs. 317, LTB_4_ (E) =299 vs. 369, macrophages (S) =265 vs. 317, eosinophils (S) =265 vs. 317.

* Due to place restriction in the figures we did not use the same abbreviations for exhaled breath condensate (EBC) and induced sputum (IS) as stated in the text.


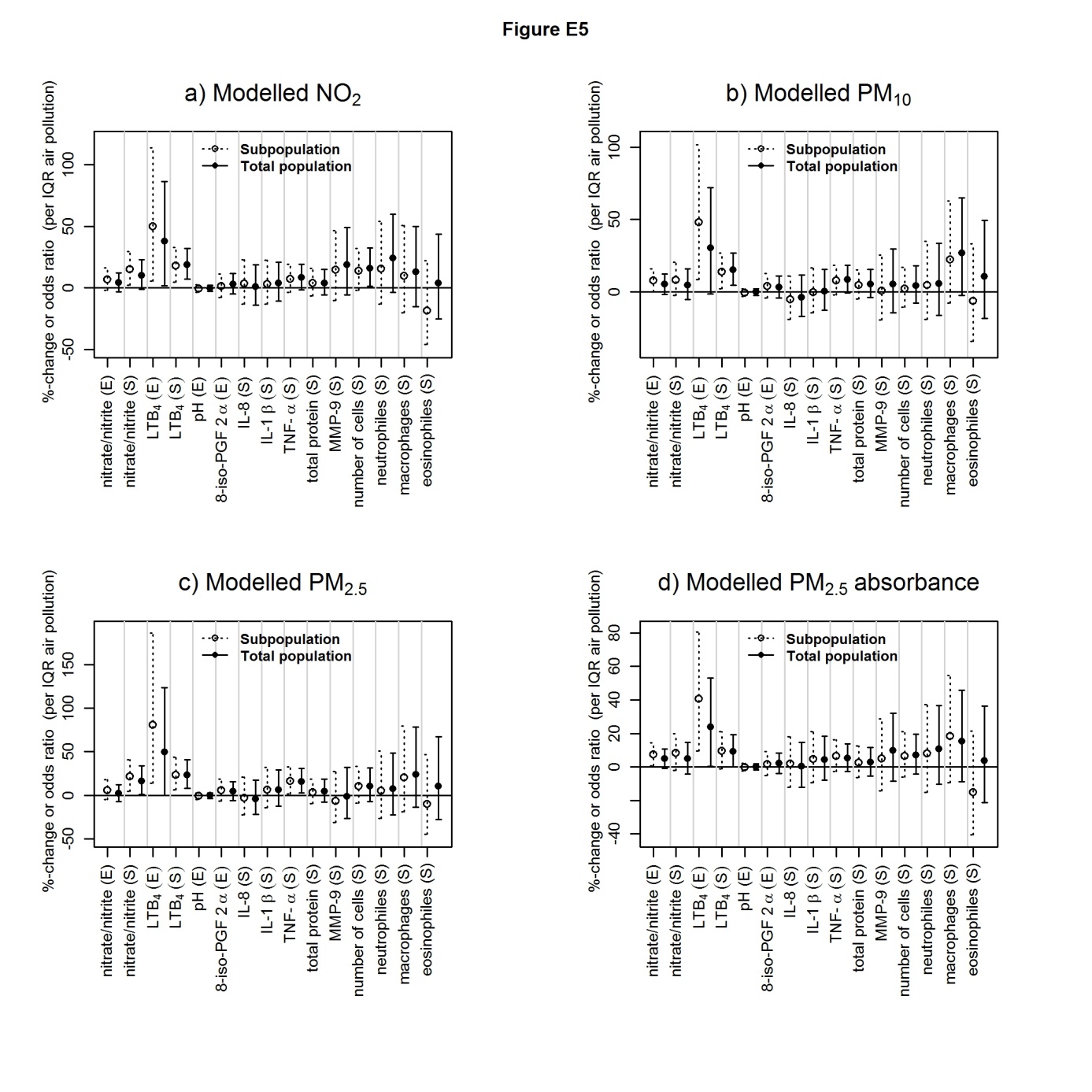
**Figure S5: Association of markers of inflammation with particles and NO_2_ (subpopulation without COPD, asthma and bronchitis vs. total population).**

Percentage changes and odds ratios with the corresponding 95% confidence intervals for inflammatory markers in exhaled breath condensate (E)* and in induced sputum (S)* for an increase by one interquartile range (IQR) of land-use regression modelled NO_2_ (a), PM_10_ (b), PM_2.5_ (c) and PM_2.5_ absorbance (d) adjusted for age, smoking (current smoking, former smoking, never smoking), current passive smoking, indoor mould and social status by years of schooling.

Number of women without respiratory diseases vs. total population for each model: NO derivatives (E) =313 vs. 380, NO derivatives (S) =270 vs. 320 , LTB_4_ (S) =270 vs. 320, pH (E) =310 vs. 377, 8-isoPGF_2α_ (E) =297 vs. 360, IL-8 (S) =264 vs. 314, IL-1β (S) =266 vs. 316, TNF-α (S) =267 vs. 316, Total protein (S) =270 vs. 320, MMP-9 (S) =233 vs. 275, number of cells (S) =270 vs. 321, neutrophils (S) =267 vs. 317, LTB_4_ (E) =305 vs. 369, macrophages (S) =267 vs. 317, eosinophils (S) =267 vs. 317.

* Due to place restriction in the figures we did not use the same abbreviations for exhaled breath condensate (EBC) and induced sputum (IS) as stated in the text.


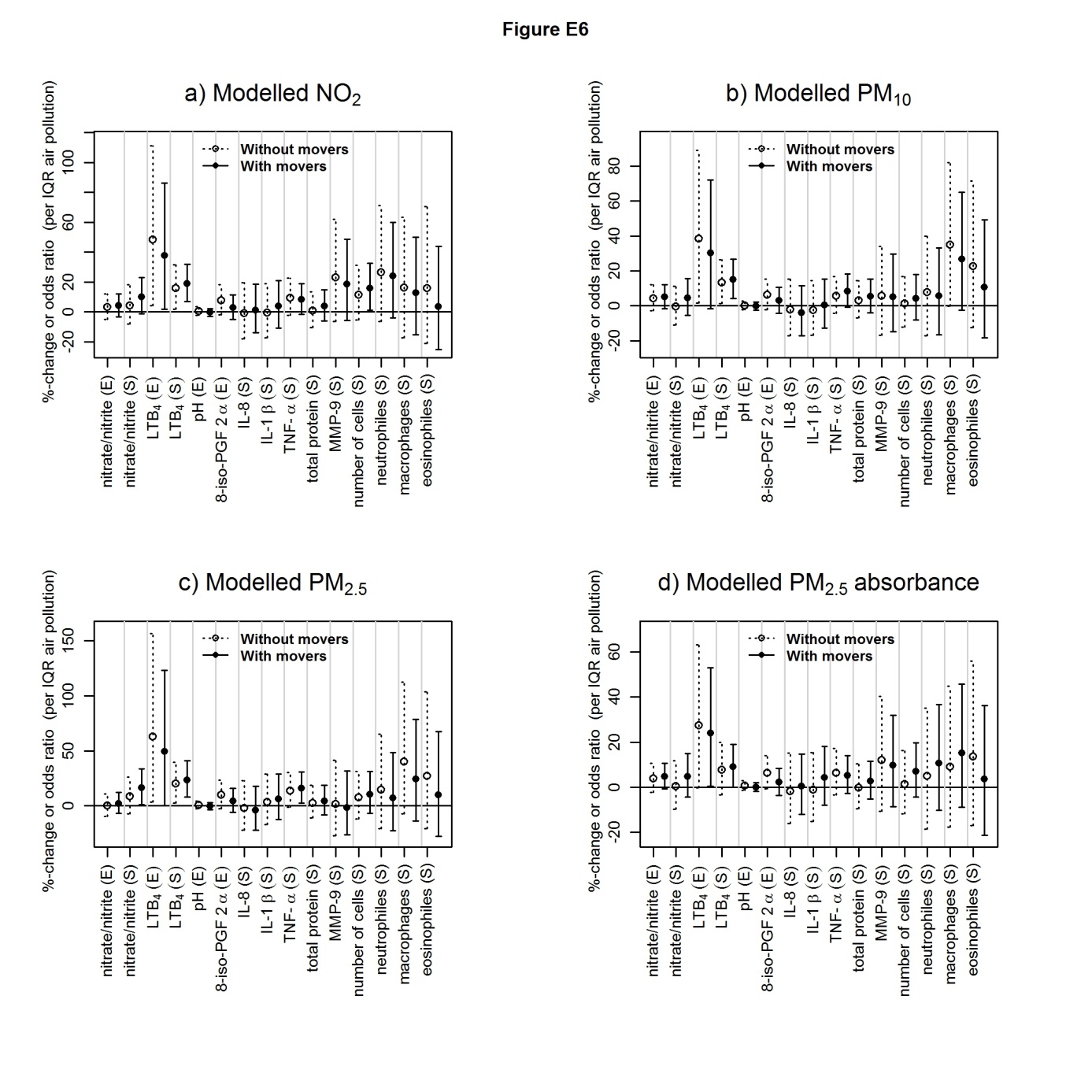
**Figure S6: Association of markers of inflammation with particles and NO_2_ (subpopulation without change of residential addresses vs. total population).**

Percentage changes and odds ratios with the corresponding 95% confidence intervals for inflammatory markers in exhaled breath condensate (E)* and in induced sputum (S)* for an increase by one interquartile range (IQR) of land-use regression modelled NO_2_ (a), PM_10_ (b), PM_2.5_ (c) and PM_2.5_ absorbance (d) adjusted for age, smoking (current smoking, former smoking, never smoking), current passive smoking, indoor mould and social status by years of schooling.

Number of women without change of residential address vs. total population for each model: NO derivatives (E) =324 vs. 380, NO derivatives (S) =275 vs. 320 , LTB_4_ (S) =275 vs. 320, pH (E) =322 vs. 377, 8-isoPGF_2α_ (E) =308 vs. 360, IL-8 (S) =269 vs. 314, IL-1β (S) =271 vs. 316, TNF-α (S) =271 vs. 316, Total protein (S) =275 vs. 320, MMP-9 (S) =234 vs. 275, number of cells (S) =275 vs. 321, neutrophils (S) =273 vs. 317, LTB_4_ (E) =316 vs. 369, macrophages (S) =273 vs. 317, eosinophils (S) =273 vs. 317.

* Due to place restriction in the figures we did not use the same abbreviations for exhaled breath condensate (EBC) and induced sputum (IS) as stated in the text.


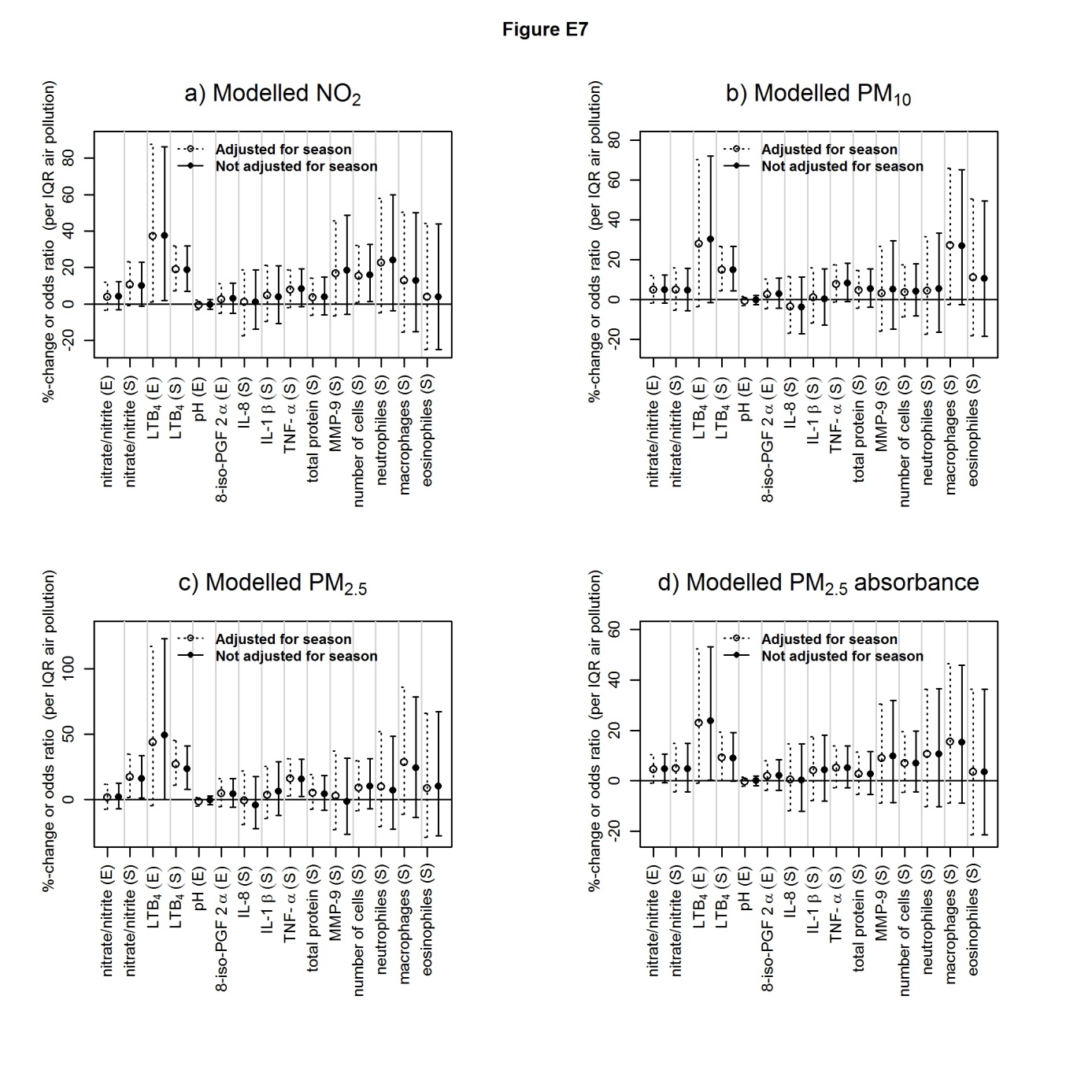
**Figure S7: Association of markers of inflammation with particles and NO_2_ (model additionally adjusted for season of examinations vs. model not adjusted for season).**

Percentage changes and odds ratios with the corresponding 95% confidence intervals for inflammatory markers in exhaled breath condensate (E)* and in induced sputum (S)* for an increase by one interquartile range (IQR) of land-use regression modelled NO_2_ (a), PM_10_ (b), PM_2.5_ (c) and PM_2.5_ absorbance (d) adjusted for age, smoking (current smoking, former smoking, never smoking), current passive smoking, indoor mould and social status by years of schooling.

Number of women for each model: NO derivatives (E) =380, NO derivatives (S) =320, LTB_4_ (S) =320, pH (E) =377, 8-isoPGF_2α_ (E) =360, IL-8 (S) =314, IL-1β (S) =316, TNF-α (S) =316, Total protein (S) =320, MMP-9 (S) =275, number of cells (S) =321, neutrophils (S) =317, LTB_4_ (E) =369, macrophages (S) =317, eosinophils (S) =317.

* Due to place restriction in the figures we did not use the same abbreviations for exhaled breath condensate (EBC) and induced sputum (IS) as stated in the text.


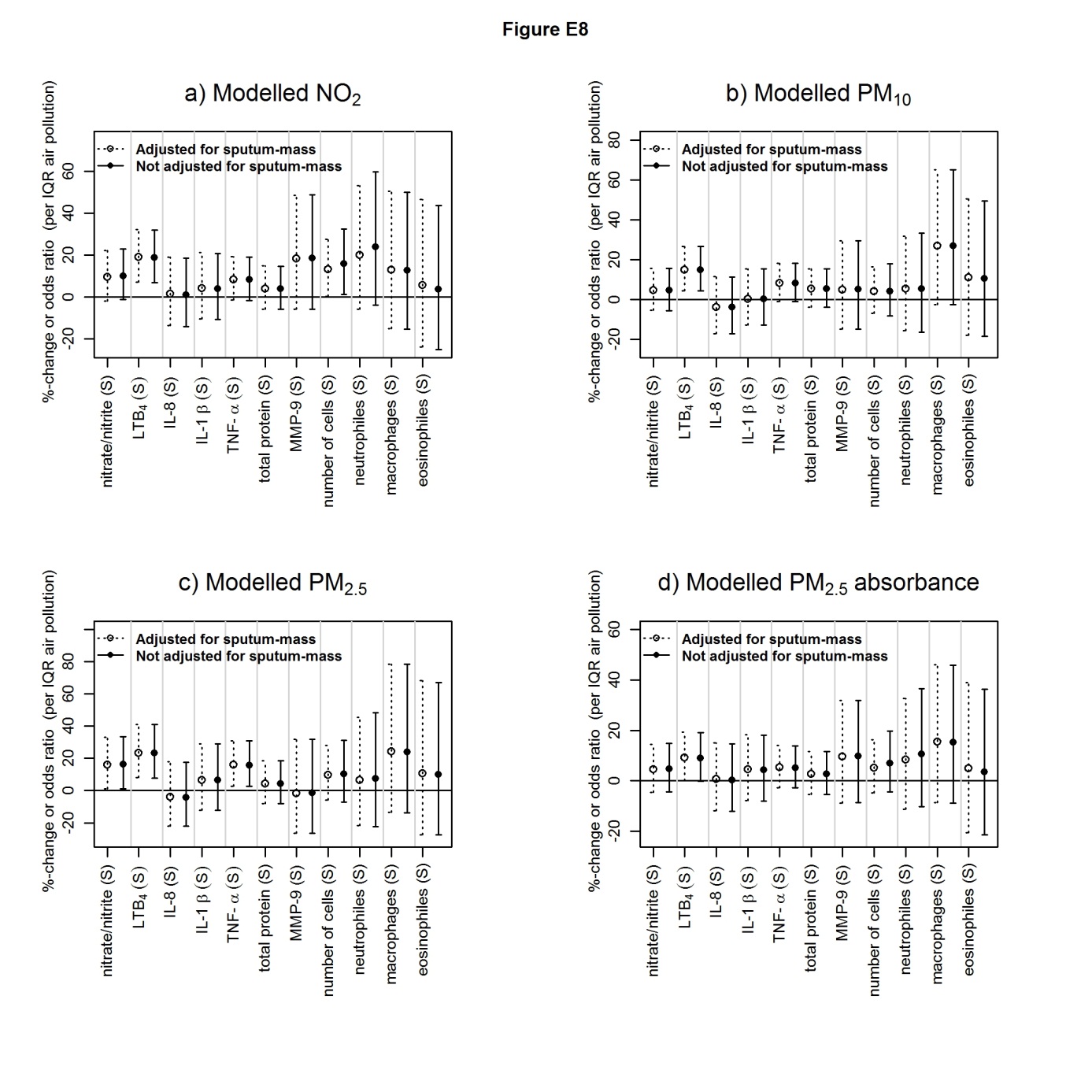
**Figure S8: Association of markers of inflammation with particles and NO_2_ (model additionally adjusted for mass of induced sputum vs. model not adjusted for mass of induced sputum).**

Percentage changes and odds ratios with the corresponding 95% confidence intervals for inflammatory markers in induced sputum (S)* for an increase by one interquartile range (IQR) of land-use regression modelled NO_2_ (a), PM_10_ (b), PM_2.5_ (c) and PM_2.5_ absorbance (d) adjusted for age, smoking (current smoking, former smoking, never smoking), current passive smoking, indoor mould and social status by years of schooling.

Number of women for each model: NO derivatives (S) =320, LTB_4_ (S) =320, IL-8 (S) =314, IL-1β (S) =316, TNF-α (S) =316, Total protein (S) =320, MMP-9 (S) =275, number of cells (S) =321, neutrophils (S) =317, macrophages (S) =317, eosinophils (S) =317.

* Due to place restriction in the figures we did not use the same abbreviations for exhaled breath condensate (EBC) and induced sputum (IS) as stated in the text.


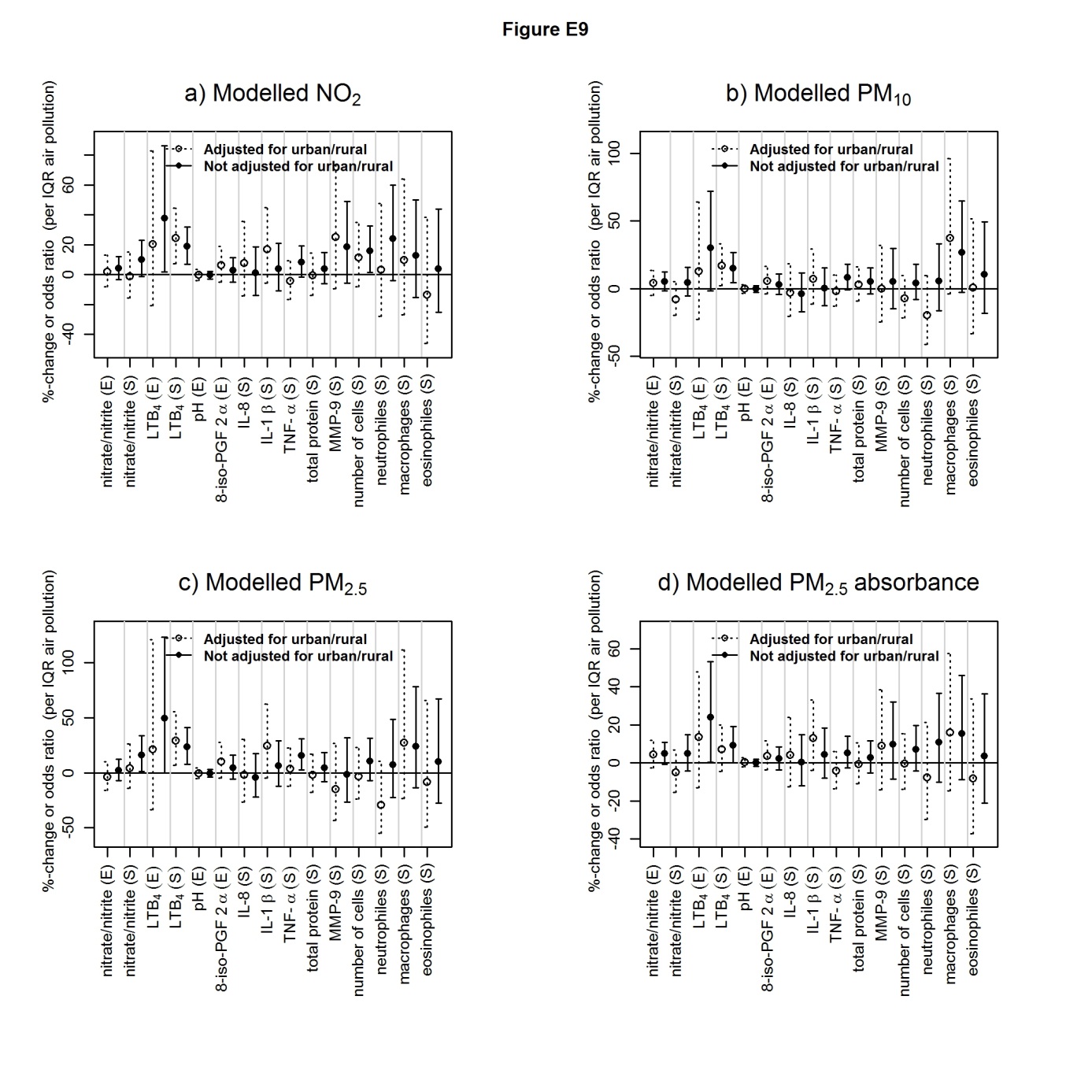
**Figure S9: Association of markers of inflammation with particles and NO_2_ (model additionally adjusted for urban/rural living vs. model not adjusted for urban/rural living).**

Percentage changes and odds ratios with the corresponding 95% confidence intervals for inflammatory markers in exhaled breath condensate (E)* and in induced sputum (S)* for an increase by one interquartile range (IQR) of land-use regression modelled NO_2_ (a), PM_10_ (b), PM_2.5_ (c) and PM_2.5_ absorbance (d) adjusted for age, smoking (current smoking, former smoking, never smoking), current passive smoking, indoor mould and social status by years of schooling.

Number of women for each model: NO derivatives (E) =380, NO derivatives (S) =320, LTB_4_ (S) =320, pH (E) =377, 8-isoPGF_2α_ (E) =360, IL-8 (S) =314, IL-1β (S) =316, TNF-α (S) =316, Total protein (S) =320, MMP-9 (S) =275, number of cells (S) =321, neutrophils (S) =317, LTB_4_ (E) =369, macrophages (S) =317, eosinophils (S) =317.

* Due to place restriction in the figures we did not use the same abbreviations for exhaled breath condensate (EBC) and induced sputum (IS) as stated in the text.


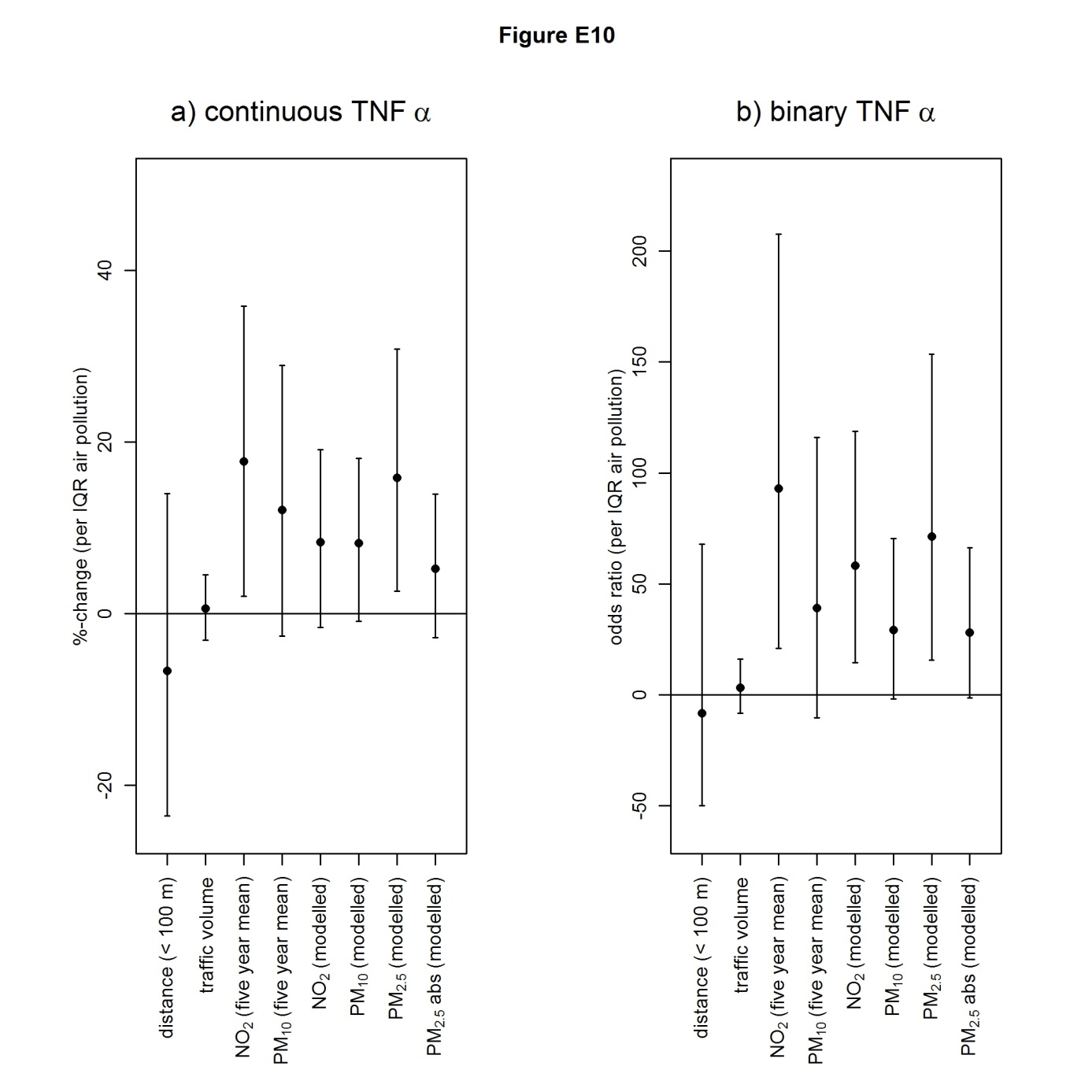


**Figure S10: Association of a) continuous variable for TNF-α and b) binary variable for TNF-α with particles, NO_2_ and traffic exposure.**

Percentage changes and odds ratios with the corresponding 95% confidence intervals for TNF-α in induced sputum for living close to major road and for an increase by one interquartile range (IQR) of traffic volume, particle and NO_2_ adjusted for age, smoking (current smoking, former smoking, never smoking), current passive smoking, indoor mould and social status by years of schooling. Number of women =316.


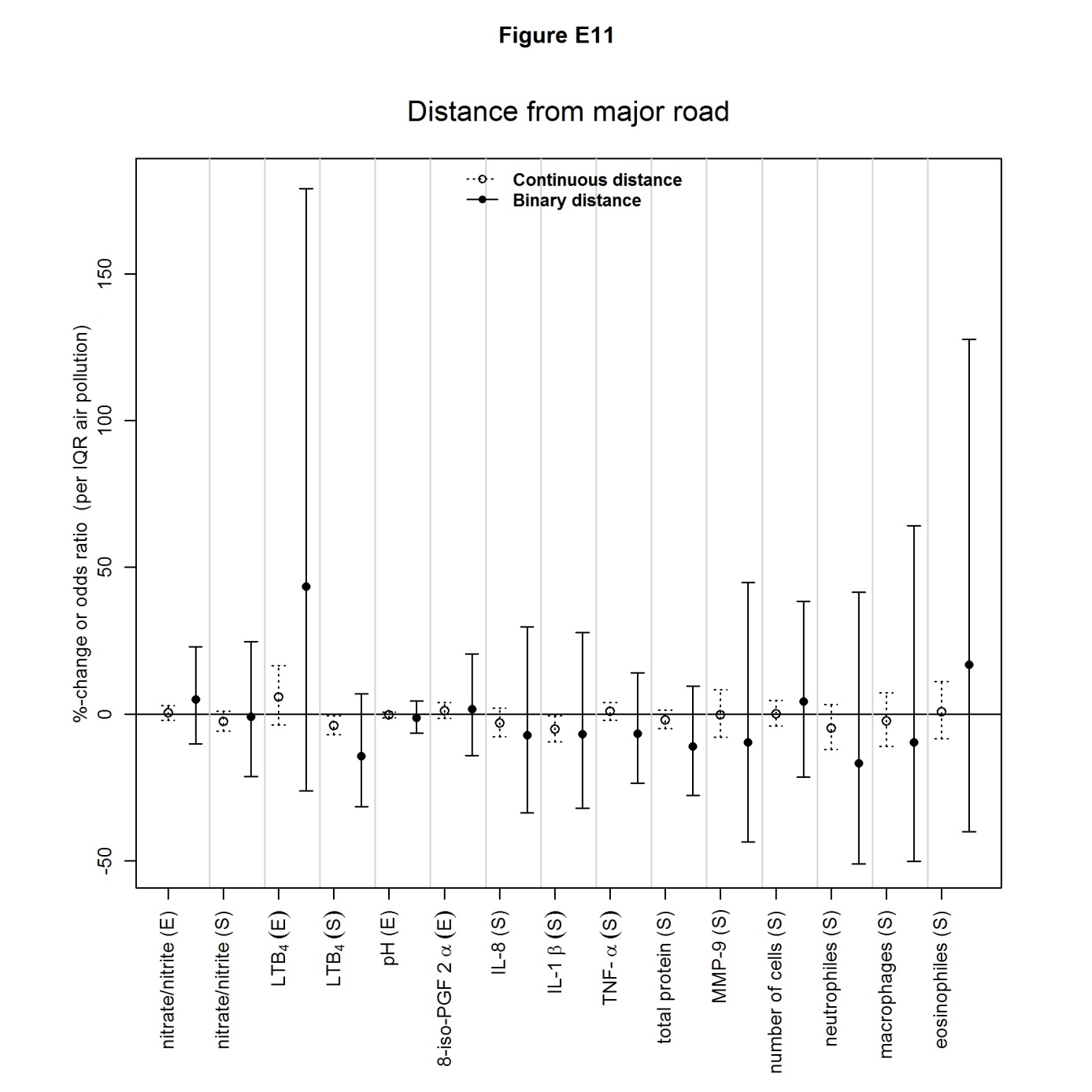


**Figure S11: Association of markers of inflammation with distance to major road (continuous variable for distance vs. binary variable for distance).**

Percentage changes and odds ratios with the corresponding 95% confidence intervals for inflammatory markers in exhaled breath condensate (E)* and in induced sputum (S)* for living close to major road as binary variable (≤ 100 m vs. > 100 m) and for an increase by one interquartile range (IQR) of continuous distance to major road adjusted for age, smoking (current smoking, former smoking, never smoking), current passive smoking, indoor mould and social status by years of schooling.

Number of women for each model: NO derivatives (E) =380, NO derivatives (S) =320, LTB_4_ (S) =320, pH (E) =377, 8-isoPGF_2α_ (E) =360, IL-8 (S) =314, IL-1β (S) =316, TNF-α (S) =316, Total protein (S) =320, MMP-9 (S) =275, number of cells (S) =321, neutrophils (S) =317, LTB_4_ (E) =369, macrophages (S) =317, eosinophils (S) =317.

* Due to place restriction in the figures we did not use the same abbreviations for exhaled breath condensate (EBC) and induced sputum (IS) as stated in the text.
